# Supplementary material for: Investigation into owner-reported differences between dogs born in versus imported into Canada
Source: PLoS One. 2022 Jun 15;17(6):e0268885. doi: 10.1371/journal.pone.0268885 (PMC9200170; doi:10.1371/journal.pone.0268885)
Supplement: S1 Appendix — (PDF) [file pone.0268885.s001.pdf]

S1 Appendix. Study 1 full survey

In the below survey, content that is in blue will not appear for the respondent.

Owner demographic information:

What is your age range?
Under 18
18-22
23-35
36-55
56-79
Above 79

What is your highest level of education completed?
Primary school/Secondary school
High school/Trade school
University education
Postgraduate education

What is your gender?
Female
Male
Other

The number of children under 18 in my household is…
(dropdown) numerical (10 or more ==10)

The number of adults 18 and over in my household is…
(dropdown) numerical (10 or more == 10)

Do you currently live with and provide care for one or more dogs?
Yes
No [if 'No' then survey discontinued]

Have you lived with and provided care to any of your dogs for more than six months?
Yes
No [if 'No' then survey discontinued]

Dog parameters:

Instructions: For the remainder of the survey, choose the youngest dog that you have provided care to for more than six months and think of them as you complete these questions.

How old is this dog?
Variable treated as numeric. Under 1 == 0.5; Over 20 == 20

Where did you get this dog from?
Purebred breeder
Through online/print/in person advertisement from stranger
Friend/relative/neighbour
Shelter/rescue/vet clinic
Pet store
Found as a stray
Offspring of another dog in my household

Did this dog come from within Canada or another country?
Within Canada
Another country

What country did this dog come from?
Sub question for 'another country' only:
Enter in blank: ______________________

Where did this dog come from as a puppy?
Born to dog from Canadian dog breeder (intentional litter)
Born to dog from international dog breeder (intentional litter)
Friend/neighbour/relative's dog had puppies
Born in animal shelter/rescue/vet clinic
Born to dog in my household
Pet store
Born to a stranger's dog (accidental litter)
Born to dog living on the streets/found/stray
Don't know

Owner-dog relationship dimensions:

Please answer the following questions based on the one dog you have already identified.

Canine behavioral assessment and research questionnaire: Excitability

Instructions:
Some dogs show little reaction to exciting events, while others become highly excited at the slightest novelty. Please indicate your own dog's recent tendency to become excitable in the following circumstances.
Scale: *0 = Calm: Little or no special reaction. 1-3 = Mild to moderate excitability. 4 = Extremely excitable: Over-reacts, hard to calm down.*

(ce1.) Just before being taken for a walk.
(ce2.) Just before being taken on a car trip.

Aggression

Instructions:

Most dogs display aggressive behaviour from time to time—e.g. barking, growling, baring teeth, snapping, etc. Please indicate your own dog's recent tendency to display aggressive behaviour in each of the following circumstances.
Scale: *0 = No aggression. No visible signs of aggression. 1-3 = Moderate aggression. Growling/barking - baring teeth. 4 = Serious aggression. Snaps, bites or attempts to bite.*

(Cag1.) When approached directly by an unfamiliar person while being walked/exercised on a leash.
(Cag2.) When toys, bones or other objects are taken away by a household member.
(Cag3.) When approached directly by a household member while s/he (the dog) is eating.
(Cag4.) When mailmen or other delivery workers approach your home.
(Cag5.) When his/her food is taken away by a household member.
(Cag6.) When approached directly by an unfamiliar dog while being walked/exercised on a leash.
(Cag7.) When strangers walk past your home when your dog is outside or in the yard.
(Cag8.) When barked, growled, or lunged at by another (unfamiliar) dog.
(Cag9.) When approached while eating by another (familiar) household dog (leave blank if no other dogs).
(Cag10.) When approached while playing with/chewing a favorite toy, bone, object, etc., by another (familiar) household dog (leave blank if no other dogs).

Fear and anxiety

Instructions:
Dogs often show signs of anxiety or fear when exposed to particular sounds, objects, persons or situations—e.g. crouching or cringing with tail tucked between the legs; whimpering or whining, freezing, trembling, or attempting to escape or hide. Please indicate your own dog's recent tendency to display fearful behaviour in the following circumstances.
Scale: *0 = no fear/anxiety: no visible signs of fear. 1-3 = mid-moderate fear/anxiety. 4 = extreme fear: cowers, retreats or hides*

(Cf1.) When approached by an unfamiliar person while away from your home.
(Cf2.) In response to sudden or loud noises (e.g. thunder, vacuum cleaner, car backfire, road drills, objects being dropped, etc.)
(Cf3.) When an unfamiliar person tries to touch or pet the dog.
(Cf4.) In response to strange or unfamiliar objects on or near the sidewalk (e.g. plastic trash bags, leaves, litter, flags flapping, etc.)
(Cf5.) When approached directly by an unfamiliar dog.
(Cf6.) When first exposed to unfamiliar situations (e.g. first car trip, first time in elevator, first visit to veterinarian, etc.)
(Cf7.) When barked, growling, or lunged at by an unfamiliar dog.
(Cf8.) When having nails clipped by a household member.
(Cf9.) When groomed or bathed by a household member.

Separation-related behaviour

Instructions:

Some dogs show signs of anxiety when left alone, even for short periods of time. Thinking back over the recent past, how often has your dog shown each of the following signs of anxiety when left, or about to be left, on its own.
Scale: *0 = Never, 1 = Seldom, 2 = Sometimes, 3 = Usually, 4 = Always*

(Cs1.) Restlessness/agitation/pacing.
(Cs2.) Barking or whining.
(Cs3.) Chewing/scratching at doors, floor, windows, curtains, etc.

Attachment and attention-seeking

Instructions:
Most dogs are strongly attached to their people, and some demand a great deal of attention and affection from them. Thinking back over the recent past, how often has your dog shown each of the following signs of attachment or attention-seeking.
Scale: *0 = Never, 1 = Seldom, 2 = Sometimes, 3 = Usually, 4 = Always*

(Catt1.) Tends to follow you (or other members of the household) about the house, from room to room.
(Catt2.) Tends to sit close to, or in contact with, you (or others) when you are sitting down.

Training and obedience

Instructions:
Some dogs are more obedient and trainable than others. Please indicate how trainable or obedient your dog has been in each of the following situations in the recent past.
Scale: *0 = Never, 1 = Seldom, 2 = Sometimes, 3 = Usually, 4 = Always*

(Ct1.) Obeys a “sit” command immediately.
(Ct2.) Obeys a “stay” command immediately.
(Ct3.) Easily distracted by interested sights, sounds, or smells.

Miscellaneous problems

Instructions:
Dogs display a wide range of miscellaneous behaviour problems in addition to those already covered. Thinking back over the recent past, please indicate how often your dog has shown any of the following behaviours.
Scale:
0 = Never, 1 = Seldom, 2 = Sometimes, 3 = Usually, 4 = Always

(Cc1.) Chases or would chase birds, given the chance.
(Cc2.) Chases or would chase squirrels, rabbits, etc., given the chance.

(Cac1.) Playful, puppyish, boisterous.
(Cac2.) Active, energetic, always on the go.

Training methods:

Instructions:

How frequently do you use the following methods to train your dog?
Scale: 4 = *At least once a day, 3 = At least once a week, 2 = At least once a month, 1 = Rarely, 0 = Never*

(T1.) I use a choke or slip collar when I walk my dog.
(T2.) I use food treats as a reward when I train my dog.
(T3.) I use an e-collar or stim collar when I train my dog.
(T4.) I use a spray bottle to train my dog.
(T5.) I alpha roll my dog.
(T6.) I give my dog verbal praise.
(T7.) I use a prong collar when I walk my dog.
(T8.) I use play as a reward when I train my dog.
(T9.) I tell my dog no when my dog misbehaves.

Dog care questions:

Instructions:
We'd like to ask you whether you agree or disagree with some very brief statements about the dog you identified.
Scale: *The following was recoded into binary: 0-1 == 0; 2-4 == 1*
0 = Strongly Disagree, 1 = Disagree, 2 = Neutral, 3 = Agree, 4 = Strongly Agree

(Dc1.) I have assistance from a dog trainer to help train this dog.
(Dc2.) I access resources from the internet to help train this dog.
(Dc3.) I use my previous experience with dogs to help train this dog.

Veterinary visit question was acquired from **Perceived health – Question 7 [H7.]**.

Lexington attachment to pets scale:

Scale:
-2 = Strongly Disagree, -1 = Disagree, 0 = Neutral, 1 = Agree, 2 = Agree Strongly

(L1.) My dog means more to me than any of my friends.
(L2.) Quite often I confide in my dog.
(L3.) I believe that dogs should have the same rights and privileges as family members.
(L4.) I believe my dog is my best friend.
(L5.) Quite often, my feelings toward people are affected by the way they react to my dog.
(L6.) I love my dog because he/she is more loyal to me than most of the people in my life.
(L7.) I enjoy showing other people pictures of my dog.
(L8.) I think my dog is just a dog.
(L9.) I love my dog because it never judges me.
(L10.) My dog knows when I'm feeling bad.
(L11.) I often talk to other people about my dog.
(L12.) My dog understands me.
(L13.) I believe that loving my dog helps me stay healthy.
(L14.) Dogs deserve as much respect as humans do.
(L15.) My dog and I have a very close relationship.

(L16.) I would do almost anything to take care of my dog.
(L17.) I play with my dog quite often.
(L18.) I consider my dog to be a great companion.
(L19.) My dog makes me feel happy.
(L20.) I feel that my dog is a part of my family.
(L21.) I am not very attached to my dog.
(L22.) Owning a dog adds to my happiness.
(L23.) I consider my dog to be a friend.

Monash dog owner relationship scale:

Scale: *-2 = Strongly Disagree, -1 = Disagree, 0 = Neutral, 1 = Agree, 2 = Strongly Agree*
(M1.) My dog costs too much money.
(M2.) My dog makes too much mess.
(M3.) There are major aspects of owning a dog I don't like.
(M4.) It bothers me that my dog stops me doing things I enjoyed before I owned it.
(M5.) It is annoying that sometimes I have to change my plans because of my dog.

Scale: *4 = At least once a day, 3 = Two to three times a week, 2 = Once a week, 1 = At least once a month, 0 = Almost never*
(M6.) How often do you feel that having a dog is more trouble than it's worth?
(M7.) How often do you feel that looking after your dog is a chore?
(M8.) How often does your dog stop you doing things you want to?

Scale: *2 = Very Hard, 1 = Hard, 0 = Neither Hard nor Easy, -1 = Easy, - 2 = Very Easy*
(M9.) How hard is it to look after your pet?

Human animal bond:

Scale: *-2 = Strongly Disagree, -1 = Disagree, 0 = Neutral, 1 = Agree, 2 = Strongly Agree*
(H1.) My dog makes me feel good about myself.
(H2.) I regret getting a dog because of all the things I have to do to care for it.
(H3.) Taking care of this dog is more work than I expected.
(H4.) I think of my dog as a member of the family.
(H5.) I have regrets about getting my dog.
(H6.) I think of my dog as a close friend.
(H7.) I regret getting my dog because of his/her behaviour problems.
(H8.) This dog has more behaviour challenges than I expected.
(H9.) Taking care of my dog is a burden.

Expectations of dog:

Scale: *-2 = Strongly Disagree, -1 = Disagree, 0 = Neutral, 1 = Agree, 2 = Strongly Agree*
(E1.) I got this dog to be a friend.
(E2.) I got this dog to do a job.
(E3.) This dog is for protecting my family.
(E4.) This dog is to provide support for my mental health.

Perceived health:

Instructions:
In the last 6 months, how often has your dog had problems with:
Scale: *4 = At least once a day, 3 = Two to three times a week, 2 = Once a week, 1 = At least once a month, 0 = Almost never*

(H1.) My dog has difficulty breathing.
(H2.) My dog has difficulty getting up after lying down.
(H3.) My dog has physical problems going for walks.
(H4.) My dog chews or scratches certain areas until it is red or irritated.

(H5.) My dog has external parasites (e.g. fleas/ticks).
(H6.) My dog has internal parasites (e.g. worms in stool).
(H7.) My dog has had a veterinary visit within the last year.

Medical cost:

In the last year, approximately how much did you spend on your dog's total medical expenses (excluding food)?
Numerical
