# Supplementary material for: Investigation into owner-reported differences between dogs born in versus imported into Canada
Source: PLoS One. 2022 Jun 15;17(6):e0268885. doi: 10.1371/journal.pone.0268885 (PMC9200170; doi:10.1371/journal.pone.0268885)
Supplement: S2 Appendix — (PDF) [file pone.0268885.s002.pdf]

S2 Appendix. Study 2 full survey

In the below survey, content that is in blue will not appear for the respondent.

Owner demographic information:

In which province do you live? [Terminate if not British Columbia]
Enter Blank: ____________

Is your gender male or female or do you identify your gender in other terms?
Male
Female
Non-binary
Other terms

Please indicate your year of birth.
Numerical slider

Do you or anyone else in your household own a dog or cat?
Currently have at least one dog
Currently have at least one cat
Currently have at least one of both
No

[If 'No' for previous question]
Have you or anyone else in your household owned a dog or cat in the last 5 years?
Yes, had at least one dog in last 5 years
Yes, had at least one cat in last 5 years
Yes, had at least one of both in last 5 years
No [If 'No', Terminate]

[If currently an owner of a dog or a cat]
How many dogs and/or cats do you currently have? [SINGLE CHOICE GRID]

	0	1	2	3	4	5 or more
Dog						
Cat						

Have you or anyone else in your household acquired a cat or dog during the COVID-19 pandemic (since April 2020)?
Yes – cat(s)
Yes – dog(s)
Yes - both
No

Dog parameters:

[Following section displayed only for current or previous dog owners]

Instructions:

The next group of questions is about your dog(s). If you have multiple dogs in your household, please choose one dog and think of them as you complete these questions. Similarly, if you currently do not have dogs, but used to have multiple dogs within the past 5 years, please think of just one of those dogs for these questions.

Was your dog born in Canada?

Yes
No
Don't know

[If 'No' for previous question]

Since your dog was born outside of Canada, which situation best describes how your dog got into Canada?

I brought my dog with me when I moved to Canada
I purchased my dog from a foreign breeder
I worked with a rescue or animal shelter to adopt a foreign dog
I got my dog from someone else who had brought the dog to Canada
Other (my dog came into Canada for a different reason)
Not applicable (my dog was born in Canada)

Is your dog a purebred (including intentional breed crosses, such as "Goldendoodle", "Cockapoo", etc.)?

Yes
No, it is a mixed breed
Don't know

[If 'Yes' For previous question]

What breed is your dog?

Labrador retriever
Golden retriever
Shih Tzu
German shepherd
Chihuahua
Goldendoodle
Yorkshire terrier
Poodle
French bulldog
Dachshund
Siberian husky
Border collie
Labradoodle

Beagle
Pug
Australian Shepherd
Boxer
Pomeranian
Cockapoo
Other (Specify)
Don't know

How big is your dog? If your dog is not yet an adult, please estimate your dog's adult size.
Small *(under 10 kg – similar to Chihuahua, French Bulldog, Shiba Inu, Toy and Miniature Poodle)*
Medium *(between 10 and 20 kg – similar to Border Collie, Cocker Spaniel, English Bulldog, Beagle)*
Large *(over 20 kg – similar to Labrador Retriever, Doberman Pincher, Standard Poodle, German Shepherd)*

How old was your dog when it came into your household?
Newborn (less than 8 weeks of age)
Puppy (8 weeks to under 5 months)
Adolescent (5 months to under 1 year)
Adult (1 year to under 8 years)
Senior (over 8 years of age)
Don't know

Owner-dog relationship dimensions:

Instructions:
Please indicate if you agree or disagree with each of the following statements.
Scale: *0= I don't know, 1 = Strongly Disagree, 2 = Disagree, 3 = Neutral, 4 = Agree, 5 = Strongly agree*

[Order Randomized]
My dog has challenging behaviour

It bothers me that my dog stops me doing things I enjoyed before I owned it

My dog and I have a very close relationship

I only use positive reinforcement methods to train my dog

My dog has no health problems
