# Supplementary material for: Investigation into owner-reported differences between dogs born in versus imported into Canada
Source: PLoS One. 2022 Jun 15;17(6):e0268885. doi: 10.1371/journal.pone.0268885 (PMC9200170; doi:10.1371/journal.pone.0268885)
Supplement: S1 File — (DOCX) [file pone.0268885.s003.docx]

**S1 Supporting information. Study 1 summary of survey components**

**Owner demographic information:**

This section contained five questions regarding the owner or their household environment: owner age group, highest level of education completed, gender, and the number of children (under 18) and adults in the household. Questions regarding the age group, education, and gender generated categorical data. The questions regarding the number of children and adults in the household generated continuous data.

**Dog parameters:**

This section contained six questions about the dog: age, country of origin, dog source (from where the owner got their dog), puppy source (from where the dog came as a puppy), international rescue (whether the dog was rescued from a foreign country), and medical cost (approximate medical expenses spent in the last year). International rescue was a new variable created using the owner responses. International rescue was coded “Yes” if owners of non-Canadian dogs also answered either “Shelter/rescue/vet clinic”, “Found as a stray”, or “Through online/print/in person advertisement from stranger” in the dog source question. Anything else was coded “No”. Responses for dog age and medical cost generated continuous data. The remaining questions generated categorical data.

#### Perceived behavioural problems:

A shortened version of the Canine Behavioral Assessment and Research Questionnaire (C-BARQ) was used to investigate the common behavioural problems encountered by BC dog owners. The shortened C-BARQ was used in this study and consisted of 31 questions exploring canine behavioural tendencies such as excitability, aggression, fear and anxiety, and separation-related behaviours, among others. Responses were collected using a 5-point scale indicating either the intensity of the behaviour (0 = No visible sign of behaviour, 1~3 = Mild to moderate sign of behaviour, 4 = Extreme display of behaviour) or the likelihood of the behaviour occurring (0 = Never, 1 = Seldom, 2 = Sometimes, 3 = Usually, 4 = Always). Two questions from the Attachment & attention-seeking section were omitted due to human error, totaling to 29 C-BARQ questions in the questionnaire.

**Perceived cost of care:**

The Monash Dog Owner Relationship Scale (MDORS) is a 28-item questionnaire that measures the owner’s perception of the dog-owner relationship using a 5-point Likert scale (-2 = Strongly disagree, -1 = Disagree, 0 = Neutral, 1 = Agree, 2 = Strongly agree) or a 5-point scale measuring the frequency of a specified activity (4 = At least once a day, 3 = Two to three times a week, 2 = Once a week, 1 = At least once a month, 0 = Almost never). The MDORS has 3 subscales which are: owner-dog interaction, perceived emotional closeness, and perceived cost. The current study utilized only the questions from the ‘perceived cost’ subscale, resulting in a total of 9 questions. These questions allowed the investigation of owner-perceived level of burden of dog ownership.

**Satisfaction with their dog:**

The owner’s satisfaction with their dog was evaluated using questions from the Human-Animal Bond (HAB) questionnaire. In its full form, this questionnaire contains 41 items; in the current study, questions were extracted from the original HAB questionnaire (mainly from the ‘no regrets’ subscale) to quantify owner satisfaction with their canine companion. Responses were scored on a 5-point scale (-2 = Strongly disagree, -1 = Disagree, 0 = Neutral, 1 = Agree, 2 = Strongly agree).

**Training methods:**

The training methods used by owners were measured using a set of 9 questions. The questions asked how often various training methods were used on a 5-point scale (0 = Never, 1 = Rarely, 2 = At least once a month, 3 = At least once a week, 4 = At least once a day).

**Perceived health:**

Perceived health of the dog was measured using 4 questions. Each question described a health problem, with responses indicating the frequency of its occurrence in the last 6 months using a 5-point scale (0 = Almost never, 1 = At least once a month, 2 = Once a week, 3 = Two to three times a week, 4 = At least once a day).

**Expectation:**

The owner’s expectation of their dog was measured using 4 questions. Each question described a reason for obtaining a dog, and a 5-point scale was used to measure the owner’s agreement or disagreement with the question (-2 = Strongly disagree, -1 = Disagree, 0 = Neutral, 1 = Agree, 2 = Strongly agree).

**Attachment:**

Measurement of owner attachment to their dog was done through the use of the Lexington Attachment to Pet Scale (LAPS). This instrument contains 23 questions assessing the owner’s level of attachment to their companion animal. In this study, responses were scored on a 5-point scale (-2 = Strongly disagree, -1 = Disagree, 0 = Neutral, 1 = Agree, 2 = Strongly disagree).

**Dog care questions:**

This section contained 4 questions: whether the owner had assistance from a professional trainer when training the dog, whether the owner accessed online resources when training the dog, whether the owner used previous experience with dogs to train the dog, and whether the dog had a veterinary visit within the last year. All responses except to the veterinary visit question were collected on a 5-point scale (0 = Strongly disagree, 1 = Disagree, 2 = Neutral, 3 = Agree, 4 = Strongly agree), which was recoded into binary form, where responses 0~1 were recoded as 0 (“No”) and 2~4 were recoded as 1 (“Yes”). The veterinary visit question was collected in binary form and did not require recoding.

**Table 1**. **Dog country of origin.**

| **Country** | | **Count** | | **Percentage** | |
| --- | --- | --- | --- | --- | --- |
| Canada | | 745 | | 92.78 | |
| Abroad | | 58 | | 7.22 | |
| United States of America | | 35 | | 4.36 |  |
| South Korea | | 6 | | 0.75 |  |
| Mexico | | 4 | | 0.50 |  |
| Thailand | | 3 | | 0.37 |  |
| Taiwan | | 2 | | 0.25 |  |
| China | | 2 | | 0.25 |  |
| Russia | | 2 | | 0.25 |  |
| Ukraine | | 1 | | 0.13 |  |
| Iran | | 1 | | 0.13 |  |
| Belgium | | 1 | | 0.13 |  |
| Philippines | | 1 | | 0.13 |  |
| **Total:** | | **803** | | **100** | |

Number of responses (count) and percentage of the countries from where respondents obtained their dogs. Further breakdown of foreign-sourced dogs is presented.

**EFA factors and reliability**

**Table 2.** **C-BARQ questions, factor loadings, and internal consistency**

| Items | Factor 1: “Difficult behaviour” | Factor 2: “Excitability” |
| --- | --- | --- |
| Ce1. Excitement before walk |  | .489 |
| Ce2. Excitement before car trip |  | .399 |
| Cs1. Restlessness/agitation/pacing when left alone | .516 |  |
| Cs2. Barking/whining when left alone | .490 |  |
| Cs3. Chewing/scratching at doors/floors/etc. when left alone | .545 |  |
| Ct1. Obeys ‘sit’ command immediately |  | .304 |
| Ct2. Obeys ‘stay’ command immediately |  | .208 |
| Ct3. Easily distracted by interesting sights, sounds, or smells |  | .473 |
| Cc1. Chases birds given the chance |  | .565 |
| Cc2. Chases squirrels, rabbits, etc. when given the chance |  | .563 |
| Cac1. Playful, puppyish, boisterous |  | .717 |
| Cac2. Active, energetic, always on the go |  | .699 |
| Cag1. Aggression when approached by an unfamiliar person while walking on leash | .725 |  |
| Cag2. Aggression when toys or other objects are taken away by a household member | .661 |  |
| Cag3. Aggression when approached by a household member while eating | .627 |  |
| Cag4. Aggression when delivery worker approaches the home | .659 |  |
| Cag5. Aggression when food is taken away by a household member | .637 |  |
| Cag6. Aggression when approached by an unfamiliar dog while walking on leash | .661 |  |
| Cag7. Aggression when outside and strangers walk past the home | .729 |  |
| Cag8. Aggression when barked, growled, or lunged at by an unfamiliar dog | .549 |  |
| Cf1. Fear when approached by an unfamiliar person while away from the home | .695 |  |
| Cf2. Fear in response to sudden or loud noises | .488 |  |
| Cf3. Fear when an unfamiliar person tries to touch or pet | .754 |  |
| Cf4. Fear in response to unfamiliar objects on the sidewalk | .658 |  |
| Cf5. Fear when approached by an unfamiliar dog | .668 |  |
| Cf6. Fear when first exposed to unfamiliar situations | .641 |  |
| Cf7. Fear when barked, growled, or lunged at by an unfamiliar dog | .521 |  |
| Cf8. Fear when having nails clipped by a household member | .479 |  |
| Cf9. Fear when groomed or bathed by a household member | .519 |  |
| **Cronbach’s alpha** | .92 | .75 |

Factor loadings of shortened C-BARQ questions. Cronbach’s alpha values for each factor are represented under each factor. Items have been slightly reworded to shorten their length. See S1 Appendix for full detail.

**Table 3.** **MDORS questions, factor loadings, and internal consistency**

| **Items** | **Factor 1: “Struggle”** | **Factor 2: “Burden”** |
| --- | --- | --- |
| M1. My dog costs too much money | .678 |  |
| M2. My dog makes too much mess | .676 |  |
| M3. There are major aspects of owning a dog I dislike | .747 |  |
| M4. It bothers me that my dog stops me from doing things I enjoyed previously | .851 |  |
| M5. It is annoying that sometimes I have to change my plans because of my dog | .813 |  |
| M6. How often do you feel that having a dog is more trouble than it’s worth |  | .704 |
| M7. How often do you feel that looking after your dog is a chore |  | .798 |
| M8. How often does your dog stop doing things you want to do |  | .730 |
| **Cronbach’s alpha** | .89 | .82 |

Questions asked and factor loadings of MDORS questions. Cronbach’s alpha values for each factor are presented under each factor. Items have been slightly reworded to shorten their length. See S1 Appendix for full detail.

**Table 4.** **HAB questions, factor loadings, and internal consistency**

| **Items** | **Factor 1: “Regret”** | **Factor 2: “Satisfaction”** |
| --- | --- | --- |
| H1. My dog makes me feel good about myself |  | .675 |
| H2. I regret getting a dog because of all the things I have to do to care for it | .814 |  |
| H3. Taking care of this dog is more work than I expected | .623 |  |
| H4. I think of my dog as a member of the family |  | .792 |
| H5. I have regrets about getting my dog | .814 |  |
| H6. I think of my dog as a close friend |  | .829 |
| H7. I regret getting my dog because of their behaviour problems | .861 |  |
| H8. This dog has more behavioural challenges than I expected | .723 |  |
| H9. Taking care of my dog is a burden | .829 |  |
| **Cronbach’s alpha** | .91 | .83 |

Questions asked and factor loadings of HAB questions. Cronbach’s alpha values for each factor are presented under each factor. Items have been slightly reworded to shorten their length. See S1 Appendix for full detail.

**Table 5.** **Training methods questions, factor loadings, and internal consistency**

| **Items** | **Factor 1: “Harsh training”** | **Factor 2: “Gentle training”** |
| --- | --- | --- |
| T1. Choke collar | .514 |  |
| T2. Treats as reward |  | .495 |
| T3. Shock collar | .786 |  |
| T4. Water spray | .664 |  |
| T5. Alpha roll | .564 |  |
| T6. Praise as reward | -.312 | .548 |
| T7. Prong collar | .784 |  |
| T8. Play as reward |  | .487 |
| T9. Verbal reprimand |  | .505 |
| **Cronbach’s alpha** | .79 | .55 |

Items and factor loadings of Training methods questions. Cronbach’s alpha values for each factor are presented under each factor. Items have been reworded to shorten their length. See S1 Appendix for full detail.

**Table 6. Perceived health questions, factor loadings, and internal consistency**

| **Items** | **Factor 1: “Perceived health issues”** |
| --- | --- |
| H1. My dog has difficulty breathing | .717 |
| H2. My dog has difficulty getting up after lying down | .865 |
| H3. My dog has problems going for walks | .906 |
| H4. My dog chews or scratches certain areas until it is red or irritated | .658 |
| **Cronbach’s alpha** | .87 |

Questions asked and factor loading of Perceived health questions. The Cronbach’s alpha value is presented under the factor. Items have been slightly reworded to shorten their length. See S1 Appendix for full detail.

**Table 7.** **Expectation questions, factor loadings, and internal consistency**

| **Items** | **Factor 1: “Expectation”** |
| --- | --- |
| E1. I got this dog to be a friend | .371 |
| E2. This dog is for protecting my family | .782 |
| E3. I got this dog to do a job | .497 |
| E4. This dog is to provide support for my mental health | .488 |
| **Cronbach’s alpha** | .61 |

Questions asked and factor loading of Expectation questions. The Cronbach’s alpha value is presented under the factor. Items have been slightly reworded to shorten their length. See S1 Appendix for full detail.

**Table 8. LAPS questions, factor loadings, and internal consistency**

| Items | Factor 1: “Attachment” |
| --- | --- |
| L1. My dog means more to me than my friends | .588 |
| L2. Quite often I confide in my dog | .531 |
| L3. Dogs should have the same rights as family members | .640 |
| L4. My dog is my best friend | .684 |
| L5. The way people react to my dog affects my feelings towards them | .520 |
| L6. I love my dog because they are more loyal than most people in my life | .644 |
| L7. I enjoy showing other people pictures of my dog | .572 |
| L8. I think my dog is just a dog | -.385 |
| L9. I love my dog because it never judges me | .606 |
| L10. My dog knows when I’m feeling bad | .645 |
| L11. I often talk to other people about my dog | .577 |
| L12. My dog understands me | .672 |
| L13. Loving my dog helps me stay healthy | .768 |
| L14. Dogs deserve as much respect as humans do | .781 |
| L15. My dog and I have a very close relationship | .844 |
| L16. I would do almost anything to take care of my dog | .831 |
| L17. I play with my dog quite often | .700 |
| L18. I consider my dog to be a great companion | .833 |
| L19. My dog makes me feel happy | .833 |
| L20. My dog is a part of my family | .818 |
| L21. I am not very attached to my dog | -.321 |
| L22. Owning a dog adds to my happiness | .792 |
| L23. I consider my dog to be a friend | .821 |
| **Cronbach’s alpha** | .92 |

Questions asked and factor loading of LAPS questions. The Cronbach’s alpha value is presented under the factor. Items have been slightly reworded to shorten their length. See S1 Appendix for full detail.

**ODR dimensions as a function of owner and dog parameters**

**Table 9.** **Statistical significance of owner and dog parameters on both C-BARQ factors**

| **Parameter** | **Df** | **Pillai’s trace test stat** | **F** | **Num df** | **Error df** | **p** |
| --- | --- | --- | --- | --- | --- | --- |
| Age | 1 | .016 | 6.3902 | 2 | 775 | <.05* |
| Dog age | 1 | .034 | 13.8093 | 2 | 775 | <.001* |
| Adults | 1 | .003 | 1.1100 | 2 | 775 | .330 |
| Children | 1 | .001 | .2694 | 2 | 775 | .764 |
| Medical cost | 1 | .005 | 1.9980 | 2 | 775 | .136 |
| School | 3 | .019 | 2.4962 | 6 | 1552 | <.05* |
| Gender | 2 | .012 | 2.3430 | 4 | 1552 | .053 |
| Dog origin (Canadian) | 1 | .002 | .6279 | 2 | 775 | .275 |
| Dog source | 6 | .029 | 1.9042 | 12 | 1552 | <.05* |
| Puppy source | 8 | .042 | 2.0841 | 16 | 1552 | <.05* |
| International rescue | 1 | .003 | 1.2931 | 2 | 775 | .275 |

MANOVA test statistics on C-BARQ factors, against all owner and dog parameters.

*Asterisks denotes statistically significant (P<0.05) values

**Table 10.** **Statistical significance of owner and dog parameters on both MDORS factors**

| **Parameter** | **Df** | **Pillai trace test stat** | **Approx. F** | **Num df** | **Error df** | **Pr (>F)** |
| --- | --- | --- | --- | --- | --- | --- |
| Age | 1 | .037 | 14.8587 | 2 | 775 | <.001* |
| Dog age | 1 | .001 | .4606 | 2 | 775 | .631 |
| Adults | 1 | .004 | 1.6841 | 2 | 775 | .186 |
| Children | 1 | .009 | 3.3457 | 2 | 775 | <.05* |
| Medical cost | 1 | .005 | 1.8284 | 2 | 775 | .161 |
| School | 3 | .045 | 6.0012 | 6 | 1552 | <.001* |
| Gender | 2 | .025 | 4.9924 | 4 | 1552 | <.05* |
| Dog origin (Canadian) | 1 | .006 | 2.4441 | 2 | 775 | .088 |
| Dog source | 6 | .027 | 1.7489 | 12 | 1552 | .052 |
| Puppy source | 8 | .027 | 1.7489 | 16 | 1552 | .052 |
| International rescue | 1 | .005 | 2.1534 | 2 | 775 | .117 |

MANOVA test statistics on MDORS factors, against all owner and dog parameters.

*Asterisks denotes statistically significant (P<0.05) values.

**Table 11.** **Statistical significance of owner and dog parameters on both HAB factors**

| **Parameter** | **Df** | **Pillai’s trace test stat** | **Approx. F** | **Num df** | **Error df** | **Pr (>F)** |
| --- | --- | --- | --- | --- | --- | --- |
| Age | 1 | .028 | 11.5438 | 2 | 775 | <.001* |
| Dog age | 1 | .003 | 1.1860 | 2 | 775 | .306 |
| Adults | 1 | .004 | 1.4092 | 2 | 775 | .245 |
| Children | 1 | .012 | 4.8282 | 2 | 775 | <.05* |
| Medical cost | 1 | .010 | 3.9742 | 2 | 775 | <.05* |
| School | 3 | .034 | 4.4426 | 6 | 1552 | <.001* |
| Gender | 2 | .032 | 6.2145 | 4 | 1552 | <.001* |
| Dog origin (Canadian) | 1 | .006 | 2.3023 | 2 | 775 | .101 |
| Dog source | 6 | .044 | 2.9290 | 12 | 1552 | <.001* |
| Puppy source | 8 | .042 | 2.0715 | 16 | 1552 | <.05* |
| International rescue | 1 | .003 | 1.0663 | 2 | 775 | .345 |

MANOVA test statistics on HAB factors, against all owner and dog parameters.

*Asterisks denotes statistically significant (P<0.05) values.

**Table 12.** **Statistical significance of owner and dog parameters on both Training methods factors**

| **Parameter** | **Df** | **Pillai’s trace test stat** | **Approx. F** | **Num df** | **Error df** | **Pr (>F)** |
| --- | --- | --- | --- | --- | --- | --- |
| Age | 1 | .023 | 8.9792 | 2 | 775 | <.001* |
| Dog age | 1 | .035 | 14.1995 | 2 | 775 | <.001* |
| Adults | 1 | .001 | .5271 | 2 | 775 | .591 |
| Children | 1 | .013 | 5.1739 | 2 | 775 | <.05* |
| Medical cost | 1 | .006 | 2.1348 | 2 | 775 | .119 |
| School | 3 | .038 | 4.9361 | 6 | 1552 | <.001* |
| Gender | 2 | .028 | 5.5915 | 4 | 1552 | <.001* |
| Dog origin (Canadian) | 1 | .019 | 7.3376 | 2 | 775 | <.001* |
| Dog source | 6 | .049 | 3.2476 | 12 | 1552 | <.001* |
| Puppy source | 8 | .055 | 2.7273 | 16 | 1552 | <.001* |
| International rescue | 1 | .006 | 2.4875 | 2 | 775 | .084 |

MANOVA test statistics on Training methods factors, against all owner and dog parameters. *Asterisks denotes statistically significant (P<0.05) values.

**Table 13.** **Statistical significance of owner and dog parameters on Perceived health issues factor**

| **Parameter** | **Df** | **Sum Sq.** | **Mean Sq.** | **F-value** | **Pr (>F)** |
| --- | --- | --- | --- | --- | --- |
| Age | 1 | 4.44 | 4.440 | 5.418 | <.05* |
| Dog age | 1 | 25.38 | 25.377 | 30.966 | <.001* |
| Adults | 1 | 1.30 | 1.299 | 1.586 | .208 |
| Children | 1 | 7.02 | 7.021 | 8.568 | <.05* |
| Medical cost | 1 | 2.40 | 2.401 | 2.930 | .087 |
| School | 3 | 13.72 | 4.573 | 5.580 | <.001* |
| Gender | 2 | 1.11 | .556 | .678 | .508 |
| Dog origin (Canadian) | 1 | 2.20 | 2.201 | 2.686 | .102 |
| Dog source | 6 | 21.51 | 3.585 | 4.374 | <.001* |
| Puppy source | 8 | 9.26 | 1.158 | 1.413 | .187 |
| International rescue | 1 | .24 | .245 | .299 | .585 |

ANOVA test statistic on Perceived health issues factor, against all owner and dog parameters. *Asterisks denotes statistically significant (P<0.05) values.

**Table 14.** **Statistical significance of owner and dog parameters on Expectation factor**

| **Parameter** | **Df** | **Sum Sq.** | **Mean Sq.** | **F-value** | **Pr (>F)** |
| --- | --- | --- | --- | --- | --- |
| Age | 1 | 16.90 | 16.904 | 26.613 | <.001* |
| Dog age | 1 | 11.93 | 11.932 | 18.784 | <.001* |
| Adults | 1 | .66 | .659 | 1.038 | .309 |
| Children | 1 | 3.25 | 3.254 | 5.122 | <.05* |
| Medical cost | 1 | .91 | .907 | 1.428 | .232 |
| School | 3 | .98 | .326 | .513 | .674 |
| Gender | 2 | 2.00 | 1.002 | 1.577 | .207 |
| Dog origin (Canadian) | 1 | 7.88 | 7.881 | 12.407 | <.001* |
| Dog source | 6 | 7.61 | 1.269 | 1.997 | .064 |
| Puppy source | 8 | 18.13 | 2.266 | 3.567 | <.001* |
| International rescue | 1 | 1.33 | 1.328 | 2.090 | .149 |

ANOVA test statistic on Expectation factor, against all owner and dog parameters.

*Asterisks denotes statistically significant (P<0.05) values.

**Table 15.** **Statistical significance of owner and dog parameters on Attachment factor**

| **Parameter** | **Df** | **Sum Sq.** | **Mean Sq** | **F value** | **Pr (>F)** |
| --- | --- | --- | --- | --- | --- |
| Age | 1 | .31 | .306 | .333 | .564 |
| Dog age | 1 | 2.11 | 2.114 | 2.307 | .129 |
| Adults | 1 | 1.73 | 1.726 | 1.884 | .170 |
| Children | 1 | 13.37 | 13.372 | 14.595 | <.001* |
| Medical cost | 1 | .26 | .256 | .279 | .598 |
| School | 3 | 6.33 | 2.109 | 2.302 | .076 |
| Gender | 2 | 25.52 | 12.759 | 13.925 | <.001* |
| Dog origin (Canadian) | 1 | .57 | .572 | .624 | .430 |
| Dog source | 6 | 6.42 | 1.070 | 1.168 | .321 |
| Puppy source | 8 | 4.19 | .524 | .572 | .801 |
| International rescue | 1 | .40 | .403 | .440 | .507 |

ANOVA test statistic on Attachment factor, against all owner and dog parameters.

*Asterisks denotes statistically significant (P<0.05) values.
