# Supplementary material for: Investigation into owner-reported differences between dogs born in versus imported into Canada
Source: PLoS One. 2022 Jun 15;17(6):e0268885. doi: 10.1371/journal.pone.0268885 (PMC9200170; doi:10.1371/journal.pone.0268885)
Supplement: S2 File — (DOCX) [file pone.0268885.s004.docx]

**S2 Supporting information. Study 2 supporting tables**

**Table 1.** **Non-Canadian purebred dog breeds**

| **AKC Breed Group** | **Breed** | **Count** | **Percentage** |
| --- | --- | --- | --- |
| **Toy** |  | **17** | **34** |
|  | Shih tzu | 4 | 8.0 |
|  | Pug | 3 | 6.0 |
|  | Yorkshire terrier | 3 | 6.0 |
|  | Chihuahua | 2 | 4.0 |
|  | Bichon frise | 1 | 2.0 |
|  | Cockapoo* | 1 | 2.0 |
|  | Havanese | 1 | 2.0 |
|  | Maltese | 1 | 2.0 |
|  | Pekingese | 1 | 2.0 |
| **Sporting** |  | **10** | **20.0** |
|  | Cocker spaniel | 4 | 8.0 |
|  | Golden retriever | 2 | 4.0 |
|  | Labrador retriever | 2 | 4.0 |
|  | English pointer | 1 | 2.0 |
|  | Gordon setter | 1 | 2.0 |
| **Non-sporting/terrier** |  | **8** | **16.0** |
|  | Poodle | 3 | 6.0 |
|  | Boston terrier | 1 | 2.0 |
|  | Lakeland terrier | 1 | 2.0 |
|  | Miniature schnauzer | 1 | 2.0 |
|  | Shiba inu | 1 | 2.0 |
|  | Tibetan terrier | 1 | 2.0 |
| **Herding** |  | **7** | **14.0** |
|  | German shepherd | 2 | 4.0 |
|  | Australian shepherd | 1 | 2.0 |
|  | Belgian shepherd | 1 | 2.0 |
|  | Border collie | 1 | 2.0 |
|  | Puli | 1 | 2.0 |
|  | Shetland sheepdog | 1 | 2.0 |
| **Hound** |  | **3** | **6.0** |
|  | Dachshund | 1 | 2.0 |
|  | Greyhound | 1 | 2.0 |
|  | Whippet | 1 | 2.0 |
| **Working** |  | **2** | **4.0** |
|  | Mastiff | 1 | 2.0 |
|  | Great Dane | 1 | 2.0 |
| **Foundation Stock/Other** |  | **3** | **6.0** |
|  | Jindo | 1 | 2.0 |
|  | Thai ridgeback | 1 | 2.0 |
|  | Valley bulldog* | 1 | 2.0 |
|  | **Total** | **50** | **100** |

Number of responses (count) and percentage of imported purebred dog breeds based on the American Kennel Club.

*Crossbreed is not listed in the American Kennel Club. Group membership was estimated based on similar characteristics.

**Table 2.** **Further details of non-Canadian dog acquired as a “Newborn’ (<8 weeks)**

| **Items** | | | **Count** |
| --- | --- | --- | --- |
| **Source** | | | **16** |
| Brought the dog when moving to Canada | | 4 |  |
| Purchased from a foreign breeder | | 4 |  |
| Worked with a rescue or shelter to adopt | | 4 |  |
| Obtained from someone else who brought the dog to Canada | | 1 |  |
| Not applicable since the dog was born in Canada | | 1 |  |
| Other | | 2 |  |
| **Breed** | | | **16** |
| Mixed | 10 |  |  |
| Purebred (Golden retriever, Pekingese, Havanese, Border collie, Shih tzu, and Shiba inu) | 6 |  |  |
| **Dog size** | | | **16** |
| Small (<10kg) | 7 |  |  |
| Medium (10~20kg) | 5 |  |  |
| Large (>20kg) | 4 |  |  |

Number of responses (count) of source (the method in which the dog was acquired), dog breed, and dog size of non-Canadian dogs acquired as a “Newborn” (<8 weeks).
