# Supplementary material for: Investigation into owner-reported differences between dogs born in versus imported into Canada
Source: PLoS One. 2022 Jun 15;17(6):e0268885. doi: 10.1371/journal.pone.0268885 (PMC9200170; doi:10.1371/journal.pone.0268885)
Supplement: S1 Code — (PDF) [file pone.0268885.s007.pdf]

S1 Code. Study 1 and Study 2 R code

Access to R code used in Study 1 and Study 2: "Investigation into owner-reported differences between dogs born in versus imported into Canada"

https://github.com/Kaialain/Dog-Source/releases/tag/v2.0.0
