# Supplementary material for: Investigation into owner-reported differences between dogs born in versus imported into Canada
Source: PLoS One. 2022 Jun 15;17(6):e0268885. doi: 10.1371/journal.pone.0268885 (PMC9200170; doi:10.1371/journal.pone.0268885)
Supplement: Attachment — Submitted filename: Response to Reviewers.docx [file pone.0268885.s008.docx]

**Response to Reviewers**

**Manuscript: PONE-D-21_25410**

I would like to thank all reviewers for taking the time to read this manuscript and for their thoughtful suggestions. The suggestions that were made were extremely helpful in guiding the editing process and improving the overall quality of this report. Please find our comments to the suggestions below. Any edits that were to the manuscript can be found in the “Revised Manuscript with Track Changes” document.

**Reviewer #1:**
- A timely study of current interest to a wider range of audiences. However, the paper itself does not seem to specifically set up to answer the aims of the study, and throughout the methods and results of study one, it feels more like it is a study that has been set up to generally identify a wide range of demographic factors that affect a wide range of dimensions of the dog – human owner relationship. There is much emphasis on other significant variables when I presume the key reason for including these if this study was genuinely designed to answer the aims set out would be to account for potential partial confounds and / or explanatory variables in the type of people that adopt overseas dogs or characteristics of these dogs that might act as alternative explanations for any significant difference found. i.e. you have run the multivariate analysis to find out whether, when accounting for these other variables, originating location of the dog adopted (overseas versus home grown) is significant. That does not come across at all in either the methods or the results of study one. Study two links more clearly to overseas dogs, but the quality of methodological reporting and claims made about replicating need revisiting.

*Thank you for the comment. We decided to include as much of our findings into the original manuscript as possible as there is currently limited research available in this field. However, we understand how this may have been a source of confusion and frustration for the readers. The manuscript has been edited to remove less pertinent information and instead, to only present information directly relevant to dog importation.*

- I cannot recommend this paper for acceptance without a major overhaul and rewrite. It is just far too much information, far too long, and far too unfocused at present. I think that the author has some interesting findings and the underpinning study has merit so it is worth persisting with this but it needs considerable revision.

*Thank you. Significant efforts have been made to reduce the length of the manuscript as much as possible. Less relevant tables and figures has been removed or relocated, and wording has been revised. We hope this improves the readability and focus of the report.*

- I also have major concerns about the sampling overlap between the two studies which I don’t think the author addresses through their reporting. If study 2 replicates and extends, why not just write up study two and accept that in study one you should have asked some key demographic information but failed to do so? Therefore it was necessary to repeat the study, and then treat study A as behind the scenes preliminary work (or a different paper with different aims – I am not entirely convinced this study was set up to specifically address the aims claimed over it). Or, did study B not replicate? The results suggests it might not have.

*Thank you for the comment. Study 2 was not a replication of Study 1. Study 1 investigated a broader spectrum of questions related to dog importation. The aim of Study 2 was to confirm our results from Study 1 and provide additional information.*

**Abstract**
- Clear abstract that contains all key components.

*Great, thank you!*

**Introduction**
- Line 68: “incomings” reads oddly. Perhaps consider: “imported”.

*Thank you for spotting the error. This section of the introduction was revised; thus, the suggestion is no longer applicable. Corresponding section can be found in line 65-69.*

- Line 102 – 104. I find this very confusing having an aim embedded in the middle of the introduction. Aims belong at the end of an introduction, not early in the process when you are setting the scene/providing the background to the work. Don’t make someone have to read your full introduction to appraise your research study – they often don’t have time.

*This is a great point. We agree that this formatting may be a source of confusion and frustration. Changes were made to relocate the aim to the end of the introduction section (line 138-140).*

- Lines 105 – 18: Would be useful to a citation to support each of these measurement tools, ideally the citation that links back to the seminal paper for this.

*Thank you for this suggestion. Citations were added for each measurement tool (line 131-134).*

- Overall, while interesting, I felt that this introduction was too long and felt a bit like an awkward blend of a literature review and an introduction. There is too much detail on each of the scales that the authors propose to use. I am estimating this introduction is about 1500 words long. I would estimate that cutting this down to around 750 – 1000 words would result in a much more tightly presented and succinct introduction.

*Thank you for your comment. The introduction has been revised to significantly reduce the length (from ~1700 words to 956 words).*

- Line 180: I am not convinced at this stage that you can demonstrate this is a separate set of respondents.

*Thank you for the suggestion. While we agree that we cannot show definitively that not a single participant took both surveys, however this is highly unlikely as the studies used different surveying platforms and were in different time periods. We clarified this point in the manuscript (line 1222-1225).*

**Materials & methods**
- Would be useful to see the methods (and results) reported in conjunction with CHERRIES reporting guidelines for online surveys. This would help with ensuring comprehensiveness of reporting.

*Thank you for sharing such a wonderful resource. The CHERRIES guideline was reviewed, and changes were made to the manuscript to follow the guideline to the extent possible. Since the complete unique site visitor and view rate statistics were not available, the participation rate was not reported in the revised manuscript. However, the recruitment and response rate section was revised to adhere to the CHERRIES guideline as much as possible. Response rates are mentioned at the start of the Results section (line 381-384, and 1269-1274).*

- I find the division of material into the sub-headings and the subheadings themselves quite confusing. Some of this is probably down to lack of numbering of headings and sub-headings so that it is not clearly signposted what is a sub-sub-section of which sub-section and which is not. However, I also find the allocation of content to each section sometimes confusing. For example, study 1, methods starts with a collection of detailed information which is a mix of population, survey design, and ethical review. A more conventional restructure of the methods would make this much easier to follow.

*Thank you for your suggestion. The structure of the Methods section was reworked to reduce confusion and improve the flow. The Methods sections now include four sub-headings for Study 1: Survey design, Recruitment and respondents, Statistical preparation, and Statistical analysis (line 150-377). Study 2 contains three sub-headings: Survey design, Recruitment and respondents, and Statistical analysis (line 1194-1240).*

- As a paid for panel was utilised, I assume that you know the number of individuals approached and therefore can calculate a response rate? What determined the number of people originally approached? Was this based on a sample size calculation? What company was used for the paid panel of online respondents? What was the wider demographic beyond age/education/gender? E.g. does the company only store details of dog owners, only store details of dog owners who do agility, etc or was it a much more disparate, not necessarily dog focused population that were sampled? I suspect the latter but this information needs to be provided.

*Recruitment information was retrieved to the extent possible. Unfortunately, not all statistics regarding recruitment was available, since participant invitations were sent by the survey companies (Study 1: SurveyGizmo, now titled Alchemer, Study 2: Stratcom) line 272-280, 381-384, 1220-1227, and 1269-1274.*

- Using CHERRIES will give you more clues as to the level of additional detail I would like to see for the methods, but things like how many pages long was the survey, how many questions did it include, etc would be useful as part of the methods rather than just referring the reader to the appendices. Separating out the survey sections info from the scales used means that I am unable to visualise easily the survey design and how this fits together with the scales. Of course, I can jump across to the appendices to do this but it would be much kinder on the reader if the way that the methods are reported allows the reader to visualise the broad key structure from the methodological section. The appendices are to allow the reader to look for very specific additional information, but the methods should be sufficient for the reader to be able to visual the study design. From the way currently presented, I cannot do this.

*Thank you for your suggestion. However, we would like to respectfully disagree with your suggestion as the manuscript was already lengthy, we have included both surveys into the Appendices to reduce the length.*

- Statistical reporting – from what you have reported this seems reasonable however, the big issue here for me is that I have read the statistics section several times now and I cannot clearly see from this how you are answering your research question. Obviously you are, but it is not being communicated clearly in a way that lets the reader see this. I assume your most important variable of interest is: was the owned dog imported from overseas? Yes / no, given your research question/focus but that is not coming across in all the detail provided in your methods section. I was left wondering whether you had plotted this as an outcome / dependent or independent measure. Basically in all the detailed reporting of what you had done, the key point of it all seems to be missing. This obviously needs to be addressed.

*Thank you for your comment. This issue may have been a result of over-reporting of the data. To minimize confusion, findings not directly related to dog importation (interactions between factors; Correlation matrix, interactions between factors and dog care questions; Multiple t-test, and interactions between dog care questions; Chi-squared test) were removed from the manuscript.*

**Study 2:**
- Line 793 onwards: so what measures were taken to screen out those from the panel that had already completed the survey the first time? How many people made up the panel population that were eligible and, of these, what % were approached? i.e. was there considerable overlap in the sample between study 1 and 2?

*There were no specific procedures to minimize overlap between the two studies. While it is unlikely that there is considerable overlap of participants approached between Study 1 and Study 2 since two different surveying platforms were used, there is no way to confirm this theory. This point is also included in the methods section for Study 2 of the manuscript (line 1222-1225).*

- I am confused. This study seemed to be to replicate/extend study 1’s methods/results, but I see no information about the scales and these are not reported. My concerns more generally about methods reporting remain for this study too.

*Thank you for pointing out your confusion. Reporting of the scales and measurements used in Study 1 and Study 2 were revamped to improve clarity (line 155-269, and 1201-1212). The confusion may have also been partly due to the over-reporting, which should no longer be an issue.*

- Response rates, excluded responses, etc are normally reported at the start of the results.

*Thank you for this input. Details of response rates were included at the start of Results section for Study 1 (line 381-384) and Study 2 (line 1269-1274).*

**Results**
- Line 265: response rate? How many responses were not included in the survey? Why?

*Thank you for this comment. Details of response rates were included at the start of Results section for Study 1 (line 381-384) and Study 2 (line 1269-1274).*

- Line 265: “51.7%”. Please don’t start a sentence with a numeral. This sentence should be restructured. E.g. “Approximately half (51.7%)”.

*Thank you for pointing out this error. Appropriate changes were made to the sentence (line 384).*

- This is a lot of tables to report demographic information, it might be worth merging into one table per study?

*Thank you for the suggestion. While we have taken this into consideration, we have decided to keep all tables for demographic information as they were relevant to dog importation. Instead, tables in the Results section that were less pertinent to dog importation were removed to reduce the length of the manuscript.*

- I am confused by the results material given over to reporting the factor analysis process and outcomes. This was described as part of the statistical preparation so I assumed this was behind the scenes methods designed to condense the data ready for inferential analyses. However, it is now being treated like results in an off itself. I think it is appropriate to report how questions loaded, etc onto dimensions – but not as results. It is methods, or, possibly supplementary material. Here in the results section it just detracts from answering the central aims of your study – which should have been answered immediately following reporting your sample size/response rate, and demographic data.

*Thank you for your idea. The factor analysis tables were removed from the manuscript file and relocated into the Supplementary information document.*

- Some of the Cronbach alphas for the data are very low e.g. 0.61 (line 329) and 0.55 (line 319) with questionable or poor consistency. This does not seem to have been noted or measures taken to try to improve the CA (e.g. remove an item and re-run the analysis).

*Great point. Reasoning behind inclusion of “Expectaton” and “Gentle training” factors despite low internal consistency has been elaborated in the Methods section (line 318-340).*

- Again, line 340 onwards. Some of this is methods and improved clarity in the methods section would remove the need to report methods within the results section. Stick to reporting the results, with clearer signposting as to what aim is being answered with which section of results reporting.

*Thank you for your suggestion. Major changes were made to the results section to streamline the reporting included in the Results section. Specifically, the results section was reworked to minimize confusion by removing less pertinent information. Reporting was reduced to include only data related to dog import related (i.e., Correlation matrix, Multiple t-test, and Chi-squared results have been removed).*

- One thing that is not clear is how the aims of your study are being answered. It is lost behind a scattergun reporting of anything that is significant. A classic example of this is found on page 28 where origin was significant yet you chose to report the other significant variables first (some of them), and bury origin in the middle.

*Good point. I see the source of confusion. This should no longer be an issue, as findings not pertinent to dog importation has been removed from the manuscript.*

- It is difficult to follow the tables e.g. table 11, to see what parameters are significant without additional information on the variable sub-categories. E.g. what does ‘Canadian’ denote? I have to try to pick it apart and make assumptions as I read through the other analyses but this is not ideal and make mean I am making unwarranted assumptions about how the data was handled.

*Thank you for highlighting this potential source of confusion. Tables included in the Results section for Study 1 has undergone major changes so this comment is no longer applicable. The newly designed tables should not cause the same confusion as the categories are labeled under their corresponding variable (Table 7-15; line 524-743).*

- Line 363: when you report a p value it is either p = X, where X is the exact probability, or p < X, where the actual probability is less than X. You have written p = < 0.001. This needs addressing here and anywhere else that this has been done.

*Great point. I was not aware of this error, and I fully agree with your comment. All incorrect reporting of P value (P=<) has been changed to either P=X or P<X.*

- Table 11: so what is the direction of any effect? I know, for example, that dog age and education level predicted C-BARQ, but how?

*Thank you for this suggestion. The directionality of the effect of owner and dog parameters on the factors should now be apparent as Mean and SD values are included in the newly designed tables (Table 7-15; line 524-743).*

**Reviewer #2**

- Overall, to summarise study 1’s results section: this is just too much results and too little focus. It needs cutting back and restructuring to focus on clearly addressing the study’s aims. There are 27 pages of results for study 1 alone!

*Thank you for your suggestion. Major changes have been made to the Results section to reduce the length and improve the focus of the study. Reporting was reduced to include only data related to dog import related (correlation matrix, multiple t-test, and chi-squared data was removed).*

**Discussion**
- Reasonable but I have not drilled down on this because, in light of my above comments, I am recommending that this paper needs a major overhaul, and the discussion will need to be reformatted in conjunction with this.

*Thank you for your comment. The discussion section has been reformatted to align with the findings presented in the Results section. The discussion now only includes material directly related to dog importation (Effect of dog origin, Pet store as source, Source as puppy, Household as source, and Effect of owner and dog parameters; line 888-1168).*

**Tables & figures**
- Far too many, it is overwhelming with content and they are often not provided with enough detail to appreciate any direction of effect.

Reviewer #2: Strong article from authors on a very important topic. One question I did have reading through the long introduction, is whether the study could be split into two? Just a question, would like to know the reasoning behind two studies in a single journal article.

*Thank you for this suggestion. The number of tables has been reduced to only include data directly relevant to dog importation. Additionally, the mean and standard deviation values have been added to the tables to allow for the interpretation of directionality of effect (Table 7-15; line 524-743).*

**Results:**
288: I do not know about the other reviewer, however, I would considering condensing the Cronbach’s alpha tables and section in the results to a summary and place the tables in appendix if possible. It will decrease the results section, decrease tables, but also allow readers to focus on the important information. To be fair, I went straight to ODR dimensions.

*What a wonderful idea – thank you. The EFA and Cronbach alpha tables have been relocated into the Supplementary information document to reduce the length of the Results section.*

289-302 – I think that those two paragraphs give the reader enough information on the EFA factors and reliability, review this section to add addition intext data then remove tables would be my recommendation.

*Thank you for your comment. This component has been relocated to the Methods section to improve the structure of the manuscript (line 316-344). Additionally, EFA tables have been removed and relocated to the Supplementary information document to reduce manuscript length.*

340 - 445 Is there any way to condense the number of tables in this section? Maybe summary all significant data in a single table and place rest of tables in appendix ? Allows the readers to get to focus on text, with a quick summary table.

*Thank you for your comment. Significant efforts have been made to reduce the length of the manuscript, but we believe that the results presented in this section are too crucial to investigate the effect of source on dog welfare to remove from the manuscript. However, the wording of this section has been revised to reduce length as much as possible. Additionally, less pertinent reports originally included in the Results section (Correlation matrix, Multiple t-test, and Chi-squared figures) have been removed to reduce the length.*

446-610 I would review all tables and identify a way to condense all down

*Thank you for the suggestion. Most of this section have been removed from the manuscript entirely as they were not directly relevant to dog importation. Reporting of on Dog care questions (Vet visit, Professional help, Internet help, and Experience help) are still available as they directly examine the effect of dog origin (line 755-884).*

The paper is well written; however, all the data takes away from the story, from what you are trying to say. There is a LOT of data, and I enjoy the data however, most readers will be thrown. Before I review any further, I would like authors to firstly revise results section, and secondly find a way to either split the two studies in part 1 and part 2, or condense the paper to focus on the most significant data.

*Dear Reviewer #2, thank you for your review and kind comments. The manuscript has been edited to reduce length and to focus the reporting to dog importation. Tables and figures have been streamlined to only report data directly relevant to dog importation. Factor analysis and Cronbach alpha tables were removed from the manuscript and relocated to the Supplementary information document. Wording of the manuscript have also been revised to convey only most significant findings.*
