# Supplementary material for: Investigation into owner-reported differences between dogs born in versus imported into Canada
Source: PLoS One. 2022 Jun 15;17(6):e0268885. doi: 10.1371/journal.pone.0268885 (PMC9200170; doi:10.1371/journal.pone.0268885)
Supplement: Attachment — Submitted filename: Response to Reviewers.docx [file pone.0268885.s009.docx]

**Response to Reviewers**

**Manuscript: PONE-D-21_25410R2**

I would like to thank all reviewers for taking the time to read this manuscript and for their thoughtful suggestions. The suggestions that were made were extremely helpful in guiding the editing process and improving the overall quality of this report. Please find our comments to the suggestions below. Any edits that were to the manuscript can be found in the “Revised Manuscript with Track Changes” document.

**Abstract**

*Lines 42-43 -* not necessary to insert the statistics as long as supporting results are in the body of the paper

Thank you for your suggestion. Statistics have been removed from the abstract (line 42)

**Introduction**

*Line 85 -* italicize both Genus names

Thank you for your suggestion. Suggested changes have been made (line 95).

**Study 1**

**Methods**

*Survey Design -* Please further explain the differences between your very similarly-named factors “Dog Source” and “Puppy Source”, as well as “Household as source”, which you don’t make clear until the Discussion. The reader needs this information well in advance of the Discussion. Does it makes any sense to rename your factor “Puppy Source” to something less similar to “Dog source”- e.g., maybe “Puppy Background”? Not required if clarification is made.

Thank you for your suggestion. Further information has been added under the methods section for “Dog source” and “Puppy source” (now re-named as puppy background). Household as source was not a separate measurement, but rather a category for dog source and puppy background. We hope the newly added information will clarify the ambiguity in the measurements collected (lines 143-157).

Also, it isn’t clear to me whether a dog who was obtained in Canada an adult, but whose owner reported them to be “born to dog from international breeder (intentional litter)” would be considered a Canadian or non-Canadian dog. This may not have happened, I suppose! But this question occurred to me based on lines 524-525 in the Discussion. I think it just speaks to the need to further clarify how your demographic questions were structured. I know that you have placed a paragraph about the dog parameters in the S1 Supporting Information, but particularly for the factors that you find affect the ODR dimensions in different directions, the reader needs ‘in text’ access to the different definitions.

Thank you for highlighting this confusion. There were 13 owners of “Canadian” dogs which were “born to a dog from an international breeder (intentional litter)”. These responses were included in the analysis as “Canadian” dogs, but I understand how this may not have been clear to the reader. Further information has been added to clarify this ambiguity (lines 265-272).

As well, in the S1 Supporting Information, you use the term “international rescue” which is not referred to in the main text as a factor, so doesn’t help clarify the distinctions.

Thank you for your comment. The text describing “international rescue” was accidentally removed during previous revisions. Description of “international rescue” has been restored (lines 157-161).

*Line 134 -* what does “This component…” refer to? Do you mean “These dimensions…” or only specific dimensions? Please clarify

Thank you for your comment. The referred text has been re-worded to help clarify the confusion (line 167).

*Lines 169+ -* to support this paragraph and give the reader more clarity on the structure of the dimensions you are evaluating, I’d ask you to consider creating a new ‘definitions’ Table, for the Methods section, that lists and defines all the 11 factors to be explicitly evaluated in your upcoming analysis. This table could include a summary of the source of questions for each ODR dimension and the interpretation you give the Dimension. I am not suggesting that the details from the factor loadings belong in this table- I think putting them in the Supplementary Info as you have done makes more sense.

Thank you for your suggestion. A new definitions table has been created in the methods section (line 206). The table defines each of the ODR dimensions and lists the extracted factor from each of the dimensions.

*Line 193 -* remove typo in between sentences

Thank you for spotting this error. The typo has been removed (line 235).

**Results**

*Lines 211+* - the word “majority” appears in several places (line 221, line 225) without an article in front of it- i.e., either “a” or “the”; please check throughout text

Thank you for your comment. Texts that contain the word “majority” has been revised and corrected accordingly (lines 273, 274, 1012, 1018, and 1257).

*Table 1* - move to Appendix- relevant results are reported in text

Thank you for your suggestion. The table showing non-Canadian dog origin was moved to S1 supporting information (line 279).

*Table 2 and 4 -* Can you merge these tables for clarity? I.e., have the comparisons of ‘dog source’ for ALL 803 dogs in the study side-by-side the same sources for Non-Canadian dogs, unless there is a compelling reason not to do so. There doesn’t seem to be a need for two separate tables when the same sources are being considered.

Thank you for your suggestion – what a great idea! Tables 2 and 4 have been merged (line 311).

*Table 5 -* and all tables- be specific re: the parameter “age”- specify “Owner age”, as you do for Dog age in these tables.

Thank you for your suggestion. All tables that contained “age” has been revised and corrected to instead mention “Owner age” (lines 405, 463, 527, 569, 634, and 677).

*Line 272-277* - wording is awkward for many of the sentences describing the factors… Consider this re-writing: “Owners with…..education received, **and** male owners compared to female owners **reported higher “Struggle” scores.** **Owners of** dogs that were acquired from a pet store compared to **those** obtained…..as a puppy **also reported** higher “Struggle” scores.”

Thank you for your comment. The ambiguity in the phrasing of these sentences were a result of an attempt to reduce the length of the manuscript in earlier revisions. These texts have been revised to achieve the right balance between clarity and conciseness (lines 430-660).

*Lines 281-284* - the results regarding “Burden” here are quite confusing. This is related to the above comments regarding source of dog/ puppy background, I believe. If you clarify the demographic questions earlier this should help- however, you may need to rewrite these results sentences for further clarity.

Thank you for pointing out this confusion in the text. Your confusion is, in fact a correct interpretation of the current findings. Dog source and puppy background collected similar, yet slightly different aspect of the dog’s history, and confusingly, the effect of these measurements on the owner-dog relationship was not in agreement. Further clarification was added in the reporting of this section to aid this issue (lines 438-460, 494-501, 562-566).

*Line 307* - remove “…resulted in higher “Regret” scores.”

Thank you for spotting this error. The error has been corrected (line 501).

*Line 335 -* remove second “…scored higher”.

Thank you for spotting this typo. Typo has been corrected (line 560).

---

*Line 375* - replace “than” with “compared to”

Thank you for your suggestion. Changes have been made to the text accordingly (line 655).

*Line 377 -* add “-s” to end of “score”

Thank you for your comment. Suggested changes have been made (line 657).

*Line 414+ -* Figure 1-  (and other Figures)- P values should be placed in Figure caption OR indicated with symbols on the figures (e.g. * p<.05, **p<.01, etc.)

Thank you for your suggestion. P values have been placed in the Figure caption (lines 717, 732, and 745).

**Discussion**

*Line 474-475 -* rewrite to “…of veterinarians agreed **that they were seeing** more behavioural…”

Thank you for your suggestion. Changes were made as suggested (line 775).

*Line 519+ -* Household as source-  As indicated earlier the information from lined 522-525 needs to be presented more clearly earlier.  Because of these subtle differences in the two variables, I am struggling to make sense of the comparisons in the next paragraph.

Thank you for pointing out your confusion. As you have suggested, further clarification on “Household as source” was added earlier in the manuscript (lines 146-160).

*Line 524 -* replace “unidentical” with “nonidentical”

Thank you for your suggestion. The wording has been changed to “different” (line 827).

**Study 2**

**Methods**

**Survey Design -** refer to the S2 appendix in the text around line 652 or earlier

Thank you for your suggestion. S2 appendix was referenced in the Survey design section (line 948)

*Line 655 -* suggest rewrite as follows: "The inclusion criteria **were** identical to **those of** Study 1: **participants were required to be a current resident of British Columbia, Canada, to own** at least one dog**, and to** have owned that dog for at least six months."

Thank you for your suggestion. Changes have been made accordingly (line 968).

*Line 658 -* replace “from” with “compared to”

Thank you for your suggestion. Changes have been made to the text accordingly (line 971).

*Line 659 -* consider adding “…and were separated by X months.”

Thank you for your suggestion. However, we have decided not to make this addition to text as the manuscript has lengthened with the changes that has been made in the current revision. The main concerns that were addressed by previous reviewers were regarding the length and scope of the manuscript being too large (line 972).

**Results**

Tables need to be streamlined and/or removed. See comments below.

*Table 16 -* REMOVE -results are reported in text

Thank you for your suggestion. Table 16 has been removed (line 1022).

*Lines 688-690+ -* Why is the information regarding size of Canadian dogs and purebred dog breeds in the text, but then the data for non-Canadian dogs are presented in exquisite detail in Tables? This seems to be an imbalance. At the very least, the table info for the Canadian dogs could be placed in the Appendix/SI. However, I believe a far better way to handle these differences could be one large table that makes Canadian vs. Non-Canadian dog comparisons on some (or all?) of the factors you wish to highlight. Currently, the presentation of data for non-Canadian dogs occurs in too much detail that is interesting as Supplementary Information, perhaps, but not always germane to your take-home message from these data.

Thank you for this suggestion. I see your point and agree that the tables provide interesting data but may not be as relevant to the main point with just non-Canadian data. I have taken your advice and created new tables that shows both Canadian and non-Canadian data (lines 1023-1071).

*Tables 17, 18 -* REMOVE/Move- data could be further presented in text, OR combined into single comparative table as suggested above, OR placed in SI

Thank you for your suggestion. Both tables 17 and 18 have been modified to include both Canadian and non-Canadian data (lines 1023-1065).

*Table 19 -* Move to SI only

Thank you for your suggestion. Table 19 have been moved to S2 Supporting Information (line 1070).

*Tables 20 & 21-* present as part of a single comparative table as suggested above OR in SI

Thank you for your suggestion. Table 20 (now table 17) have been modified to include both Canadian and non-Canadian data (line 1071). However, suggested changes were not able to be implemented for table 21 (now table 18) since only owners of non-Canadian dogs were asked for their dog source method. As a result, dog source methods for Canadian dogs are unavailable in Study 2. Additionally, while table 21 only shows non-Canadian dog source methods, we believe the information presented in this table are pertinent to the main interest of this research. Therefore, we have decided to keep this table in the main body of the paper (line 1100).

*Table 22 -* Move to SI only

Thank you for your suggestion. Table 22 have been moved to S2 Supporting information (line 1112).

*Line 742-743 -* rewrite: “Canadian dogs were **comprised of** 31.1% large dogs **compared to non-Canadian dogs, of which 20.7% were large dogs.**”

Thank you for your suggestion. Suggested changes have been made to the text (line 1126).

*Line 745 -* Add “-s” to “parameter” in Table 23 title

Thank you for your suggestion. Suggested changes have been made (line 1128).

*Figure 4 -* Is it possible to indicate on the figure (eg., with bolding or otherwise) which of the factors has significant ORs?

Thank you for your suggestion. Asterisks have been added next to the statistically significant independent variables to assist visibility to readers. Figure legend has also been changed accordingly (line 1150)

**Limitations**

*Line 853 -* change “was” to “were”

Thank you for your comment. Changes were made to the text accordingly (line 1245).

In this section, it may be worth considering the limitations of restricting the study to residents of BC only, as owners in other provinces may show different patterns of obtaining international dogs that are quite specific. E.g., in Newfoundland and Labrador, there is a local group that imports retired racing greyhounds from the US, and one that brings in ‘saluki’-type/middle Eastern sight hounds from Quatar. In these cases, dogs could have very different backgrounds compared to dogs that are rescued as ‘street dogs’ from Central America, for example, which could impact the ODRs you are studying.

Thank you for your suggestion. The limitation section has been revised to include potential biases resulting from respondents being limited to BC dog owners (lines 1238-1250).

**References**

I have not provided detail on each reference, but the formatting is inconsistent with respect to case of the journal article title, in particular- so please revise accordingly to align with the journal’s author instructions. These references will be further checked by the journal editing team prior to proof production.

Thank you for your comment. The reference section has been reviewed and changes have been made to fit the PLOS ONE referencing style. To our knowledge, the only difference in the referencing style of this current paper to that of PLOS ONE, is the phrasing “Available from: URL” is instead styled as “Available: URL”. This can be changed manually if necessary. However, the change has not been made in the current version of the manuscript since the reference manager (Mendeley) overrides manual changes when references get updated.
